# User perception of quality of care in patient navigation for type 1 diabetes mellitus*

**DOI:** 10.1590/1518-8345.7350.4491

**Published:** 2025-03-14

**Authors:** Luciana Foppa, Betina Nemetz, Rosimeri de Matos, Beatriz D’Agord Schaan

**Affiliations:** 1 Hospital de Clínicas de Porto Alegre, Serviço de Enfermagem Ambulatorial, Porto Alegre, RS, Brazil; 2 Universidade Federal do Rio Grande do Sul, Escola de Enfermagem, Porto Alegre, RS, Brazil; 3 Hospital São Lucas da Pontifícia Universidade Católica do Rio Grande do Sul, Porto Alegre, RS, Brazil; 4 Universidade Federal do Rio Grande do Sul, Porto Alegre, RS, Brazil

**Keywords:** Patient Navigation, Diabetes Mellitus, Type 1, Quality of Health Care, Nursing Care, Patient-Centered Care, Remote Consultation

## Abstract

to understand the perception of individuals with type 1 diabetes mellitus regarding the quality of care provided by a patient navigation program.

descriptive study with a qualitative approach, conducted at a university hospital in southern Brazil. A total of 35 individuals with type 1 diabetes mellitus participated. Data were collected through semi-structured interviews conducted via teleconsultation. The data were submitted to thematic content analysis.

of the 35 (100%) participants, 18 (51.4%) were men, with an average age of 45 (±13) years. Two thematic categories and three main topics related to care quality emerged from data analysis: diabetes management (quality of health care provided; knowledge gained during consultations; improvement in self-care practices as a result of received care) and remote and in-person health services (availability of health services during the pandemic; desire for continued access to services provided by nurses; importance of received care).

after analyzing participants’ perceptions, it was understood that the quality of care offered by the patient navigation program is generally positive. Users requested the continuation of this care model, seeing it as an opportunity beyond the challenges and limitations imposed by treatment.

## Highlights

**(1)** The quality of care provided by the patient navigation program was positive. **(2)** Patient navigation opens new avenues for research in chronic diseases. **(3)** Remote patient navigation enabled individualized and high-quality care. **(4)** Self-care practices in type 1 diabetes can be encouraged through patient navigation.

## Introduction

In 2022, there were 8.75 million people living with Type 1 Diabetes Mellitus (T1DM) worldwide, with Brazil being the third country with the highest number of cases across all age groups^(1-2)^. T1DM is a condition requiring constant management, involving daily insulin administration, regular blood glucose monitoring, adoption of a healthy lifestyle, and regular physical exercise^(3)^. These activities demand a central role from the patient and their family, which includes diabetes education from the multidisciplinary team, as well as emotional support and adherence to continuous treatment^(4-5)^. Health systems must support, prepare, and assist individuals affected by the disease and the professionals responsible for their treatment. Patient navigation is a personalized program designed to meet individual needs, continuously helping patients with chronic diseases that require self-care to overcome modifiable barriers to care and achieve their goals^(6)^.

Patient navigation emerged in the late 1980s in the United States to improve treatment adherence and overcome barriers to accessing oncology health services. The results of this program brought benefits in education and access to early diagnosis and treatment, as well as increased survival rates for cancer patients^(7)^. Currently, patient navigation programs are increasingly used by individuals with non-communicable chronic diseases worldwide, with mostly positive outcomes^(6)^. International studies on patient navigation programs in diabetes have investigated how this care model can improve disease management, patient quality of life, and health outcomes^(8-10)^. However, in Brazil, patient navigation for individuals with chronic diseases is still under development, with few published studies on the subject^(10)^.

The Federal Nursing Council regulated the role of the nurse navigator through COFEn Resolution No. 735/2024. This represents a significant advancement for Brazilian nursing, recognizing the nurse navigator as a central element in integrated and coordinated patient care^(11)^. Inspired by international models but adapted to Brazilian realities, patient navigation has proven to be a promising strategy to overcome barriers to healthcare access and improve clinical outcomes^(9,12)^.

Diabetes mellitus (DM) is a major risk factor for cardiovascular diseases, chronic kidney disease, lower limb amputation, and infections, with severe COVID-19 recently added to this list. It is important to note that poor glycemic control is associated with a high risk of worsening this infection, with an increased likelihood of developing acute respiratory distress syndrome, intensive care unit admission, and death^(13-14)^. Diabetes self-management behaviors are necessary to prevent these complications, aiming for good glycemic control to reduce the risk of complications and improve health outcomes^(15)^. Thus, patient-centered care is essential to help individuals with diabetes develop the self-management skills necessary to live with their disease.

It is worth noting that patient navigation programs are led by professionals with knowledge and skills in the field, mostly nurses who provide patient-centered care, helping to overcome modifiable barriers to care and achieve care goals by offering a personalized approach to meet individual needs^(6,8)^. Depending on the targeted barriers, specific tasks of navigators may include one or more of the following: disease education, health system education, removing barriers between doctor and patient, addressing other financial barriers, assisting in care coordination, and providing guidance on community resources, among others^(6)^.

Therefore, studies have been conducted to investigate which self-care barriers related to diabetes patients have been reporting^(16-17)^. Among the obstacles to diabetes self-management cited by patients are communication gaps between users and healthcare professionals, poor understanding of the disease condition, and financial difficulties^(16-17)^. Despite advances in healthcare and initiatives to improve care for individuals with chronic diseases like DM, there is still a significant gap in knowledge and implementation of patient navigation programs in Brazil. This gap highlights the need for more research on patient navigation in the country.

Understanding how patient navigation programs can be adapted and implemented in the Brazilian context is essential to improve care for individuals with DM, contributing to reducing health inequalities and improving care quality. In light of these circumstances, it is crucial to understand the perceptions of individuals with T1DM regarding the quality of care within the patient navigation program. This understanding aims to enhance the planning of actions and interventions for individuals with this condition through an integrated, patient-centered approach that considers the disease’s complexity and each patient’s unique needs.

Considering the objectives of patient navigation and the complexity of individuals with T1DM treated in tertiary care, there is a need for studies addressing the role of nurses in this program. Thus, the objective of this study was to understand the perception of individuals with T1DM regarding the quality of care provided by the patient navigation program.

## Method

### Type and place of study

This is a descriptive study with a qualitative approach, using Bardin’s thematic content analysis^(18)^, conducted at a university hospital in southern Brazil. This study followed the recommendations of the Consolidated Criteria for Reporting Qualitative Studies (COREQ)^(19)^.

In 2023, approximately 547,805 outpatient consultations were conducted at the hospital in question. Additionally, over 31,000 teleconsultations were performed^(20)^. The institution’s endocrinology outpatient clinic, where the research took place, provides care from endocrinologists, nurses, social workers, and nutritionists. This clinic is a reference for treating individuals with T1DM of various ages and serves patients from all regions of the state of Rio Grande do Sul.

### Population and participants

The population consisted of patients diagnosed with T1DM who were regularly followed at the institution’s endocrinology outpatient clinic. Participants were intentionally selected from a doctoral thesis study titled “Self-management of type 1 diabetes in adults: a person-centered approach to improving knowledge and treatment adherence in a tertiary hospital”, part of the Postgraduate Program in Endocrinology at the Federal University of Rio Grande do Sul.

To be included in the study, participants had to be over 18 years old and have a diagnosis of T1DM. Exclusion criteria included cognitive impairment, pregnancy, hearing loss, and substance dependence. The criterion of data saturation — repetition of information in interviews—was used to define the number of participants. Patients were progressively selected until data saturation was reached, resulting in 35 participants. Data saturation occurred when additional data collection did not yield new categories or relevant insights, indicating that sufficient knowledge had been gathered to answer the research question^(18)^.

### Data collection

Data collection was conducted by a single researcher, a master’s degree holder in nursing, during her doctoral studies. The researcher has experience in diabetes education. It is worth noting that the doctoral thesis addressed patient navigation as a strategy to improve diabetes self-management. Furthermore, three nurses and one nursing student were involved in the intervention. To avoid bias in interviews, participants who were followed by the other two nurses and the nursing student during the main study were interviewed by the researcher for this study. In other words, patients had no prior connection with the interviewer. Another important point is that patient navigation was developed during the COVID-19 pandemic, from January 2021 to April 2022.

Data were collected through semi-structured interviews that included open-ended questions about the care provided by nurse navigators during the study, the use of text messages, and teleconsultation and in-person services offered to T1DM patients at the institution. The following questions were asked: How did the care you received influence your self-care practices? How do you evaluate the availability of services (teleconsultation, text messages, and in-person care) that you received? Do you have any suggestions for improving the care you received? Interviews were conducted by phone at times chosen by participants, lasting an average of 21 minutes. They took place 30 days after the end of care with the nurse navigator, which occurred between January and April 2022. Calls were made from a room designated for teleconsultations at the research institution.

Finally, patient statements were recorded using a call-recording application available on the institution’s computer. Field notes were taken by the researcher during interviews using an online form created for this purpose. It should be clarified that no validation of statements included in the research report was conducted. The study included two pilot projects to verify language adequacy and question comprehension.

### Data analysis

As interviews were completed, they were transcribed verbatim and compared with information recorded during phone conversations. Transcriptions were reviewed by cross-checking field notes (online form) with recorded audio. Participants’ sociodemographic data were recorded using Microsoft Excel.

To ensure anonymity and respect ethical principles, interviews were coded as P1, P2, P3..., with increasing numbers corresponding to the chronological order of interviews. Likewise, information obtained was used solely for this study.

Based on the full transcripts of the interviews and to ensure methodological rigor, the information was subjected to Bardin’s thematic content analysis, conducted in three stages: pre-analysis (transcription, corpus formation, initial reading of interviews, and formulation of hypotheses and objectives), material exploration (data export to NVivo® version 15, data classification, information integration, thematic coding, and categorization), and treatment of results and interpretation (drawing inferences and interpreting data, as well as revisiting the theoretical framework)^(18)^. Additionally, quality criteria involving exhaustiveness, representativeness, relevance, and objectivity^(18)^ were valuable in accurately reflecting participants’ experiences and realities. As part of the analysis process, participants’ perceptions were understood based on how they expressed their experiences and how these expressions were interpreted and categorized during analysis^(18)^.

Data interpretation was conducted in light of theoretical grounding based on the effect of patient navigation on self-care, disease knowledge, and follow-up with the healthcare team^(7,10)^. The data analysis process involved organizing data through identification, coding, and category creation. Coding was performed after reading and marking interview transcriptions, highlighting words, phrases, or themes relevant to the study topic. Categories were formed by grouping codes or expressions with similar characteristics, establishing complementary relationships between data.

Subsequently, data classification was carried out to achieve text comprehension by segmenting it into emerging thematic categories through organizing significant expressions or words. Finally, classification and incorporation of information generated the main themes presented in the results. There was a careful review of the categories summarizing the content of the interviewees’ statements, requiring an in-depth intellectual effort.

### Ethical aspects

The study was approved by the institution’s ethics committee via *Plataforma Brasil*, under CAAE number 20380919800005327, adhering to the guidelines outlined in Resolution 466/2012 of the National Health Council. The researcher followed the institution’s telephone script for research invitations, which included three options for participants to send the Informed Consent Form (email, WhatsApp, or message), and the document was sent according to their preference.

## Results

Table 1 shows the sociodemographic and clinical characteristics of the 35 (100%) participants. It is noteworthy that 18 (51.4%) were men; the mean age was 45 (±13) years and 4 (11.4%) were active smokers.

**Table 1 -:** Distribution of study participants (n = 35) according to sociodemographic and clinical variables. Porto Alegre, RS, Brazil, 2022

Characteristic*	**n=35**
Age (years old)	45 ± 13
Gender	18 (51.4)
Male	17 (48.6)
Female	
Marital status	23 (65.7)
Married or fixed partner	9 (25.7)
Single	3 (8.6)
Widow	
Active smoker	4 (11.4)
Professional occupation	19 (54.2)
Paid activity	3 (8.6)
Unemployed	13 (37.2)
Retired	
Time since diagnosis	26.9 ± 11.4
Glycated hemoglobin (%)	8.0 ± 1.2
Comorbidities ^†^	9 (25.7)
Systemic arterial hypertension	6 (17.1)
Psychiatric disorders	6 (17.1)
Dyslipidemia	19 (54.2)
Retinopathy	3 (8.6)
Diabetic kidney disease	6 (17.1)
Sensory neuropathy ^‡^	2 (5.7)
Foot lesions	
Schooling	3 (8.6)
Elementary School	18 (51.4)
High School	14 (40)
Higher Education	
Pre-prandial insulin	6 (17.1)
Regular	29 (82.9)
Analogs	
Basal insulin	15 (42.9)
Neutral Protamine Hagedorn (NPH)	20 (57.1)
Analogs	

*Continuous variables are described by mean and standard deviation, and categorical variables by absolute number and percentile; ^†^More than one response was recorded for this variable; ^‡^Sensory neuropathy was considered present when documented in the medical record by the attending physician

**Figure 1 -:**
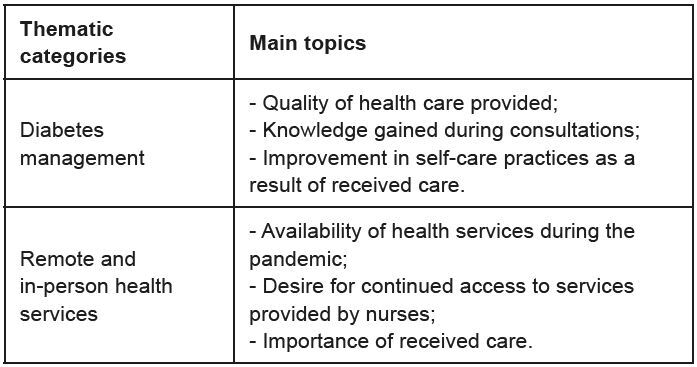
Thematic categories and main topics resulting from the analysis of participant statements (n = 35). Porto Alegre, RS, Brazil, 2022

Figure 1 presents the thematic categories and main topics that emerged from the pre-analysis and data analysis process based on the participants’ statements.

### Diabetes management

This category grouped participants’ perceptions regarding the quality of care provided, the knowledge gained during consultations, and improvements in self-care practices as a result of the received care.

Regarding the quality of care, participants reported satisfaction with the service. The interviews highlighting this theme were: P1, P2, P3, P4, P7, P11, P15, P16, P17, P20, P29, P30, P34 and P35. Most participants appreciated the offered assistance and hoped to maintain follow-up through patient navigation at the institution. The perceived quality may be strongly associated with interactions with specific professionals, such as nurses who play a central role in care. Highlighted statements include:

*Congratulations on the research, I really liked it! I really liked the nurse’s care.* (P2)

*Very good service! I would like to continue having some contact in case of doubts.* (P7)

*I really enjoyed participating in the research!* [...] *great service* [...] (P11)

The interviews that expressed opinions on the knowledge gained during consultations were: P1, P5, P6, P10, P14, P16, P18, P20, P22, P25, P26, P28 and P32. Health education plays an important role in empowering patients for self-care, preventing complications, and adhering to treatment. Some interviewees mentioned learning new foot care routines during consultations, while others referred to guidance provided on vaccines and insulin use. Highlighted excerpts include:

[...] *I really appreciated receiving guidance, especially about vaccines.* (P1)

[...] *it helped with issues that doctors had never advised me on regarding insulin application.* (P14)

*I liked learning how to moisturize my feet and elbows.* (P18)

The interviews mentioning improvement in self-care practices as a result of received care were: P9, P11, P12, P13, P18, P24, P25, P26, P27, P28 and P31. Patient navigation can be structured not only to provide information but also to prepare patients to overcome obstacles and incorporate sustainable changes. All these interviews referred to how patient navigation helped participants learn more about diabetes and its management. These conclusions emerged in the following statements:

*I thought it was very good! Very enlightening research, I started doing things I didn’t do before, maybe because of lack of knowledge* [...]. (P9)

[...] *very valuable to remember things that fall into oblivion* [...]. (P11)

*I liked learning new routines, I greatly improved my care.* (P18)

[...] *I’ve had diabetes for 31 years, and we’re always learning something.* (P31)

Given that the interviews highlighted how patient navigation significantly contributed to improving self-care practices, especially in diabetes management, it is crucial to examine how the availability and format of health services, whether remote or in-person, affect this interaction and the continuity of care.

### Remote and in-person health services

This category grouped participants’ perceptions regarding the availability of health services during the pandemic, as well as the desire for continued access to services provided by nurses and the importance of received care.

The interviews that provided information on the availability of health services to assist diabetes self-management were: P5, P8, P11, P15, P16, P19, P20, P24 and P35. These patients expressed gratitude for the availability of care they received during the COVID-19 pandemic and the accompanying social isolation measures. The pandemic posed unprecedented challenges for health systems, affecting both the supply and demand for services, particularly for people with DM, who require continuous follow-up. The following speeches stand out:

[...] *I want to thank you, I liked the care during the pandemic*. (P19)

*I found the research very good, it makes us feel more secure. Thank you for calling at night because I work during the day.* (P24)

*Thank you for the contact and saying that your care was very important, especially during the pandemic, as it was a form of follow-up during isolation.* (P35)

Participants also reported a desire for continued access to services provided by nurses after the study ended and remotely. The interviews highlighting this theme were: P2, P3, P6, P8, P11, P20, P24, P29, and P33, the statements showing interest in continuing patient navigation were: P3, P4, P7, P15, P20, P23, P29, P32 and P34. Participants’ demand for the continuation of remote services points to a possible gap in traditional health systems. These conclusions are present in the following statements:

*You should continue phone service and this differentiated attention you give; if we could continue with WhatsApp, it would be better.* (P3)

*I would like it to continue because it helped a lot with my care; it was very useful, it’s great to have someone to talk to.* (P4)

*I would like it to continue. In-person consultations are very spaced out, and this remote service brings another form of commitment, where you know you’ll be attended to and have to do things right. I really liked the online! I liked evaluating my feet!* (P20)

[...] *I think it’s essential to have a contact phone number* [...]. (P23)

The interviews that provided information on the importance of received care were: P1, P2, P5, P7, P9, P12, P13, P15, P16, P17, P22, P23, P24, P25, and P34. These interviews discussed the perception of having someone to contact in times of doubt and how much they learned from the health education provided by nurses during patient navigation. In healthcare contexts, especially for DM, continuous relationships and support offered by health professionals can be determining factors for treatment success and patient well-being. Highlighted statements include:

*I found the consultations great; I learned a lot* [...]. (P5)

[...] *I loved it; it was a very good experience; I felt more welcomed* [...]*.* (P15)

[...] *I learned a lot from the research; I found out things that doctors don’t talk about* [...]. (P25)

[...] *very grateful for the care, attention, and the education you always brought to conversations and exams.* (P34)

However, one interviewee expressed dissatisfaction with remote care and another with the interval between consultations. While remote care emerged as a viable solution to maintain patient follow-up during social distancing, it may not equally meet everyone’s needs. Additionally, the interval between consultations can influence not only satisfaction with the service but also the effectiveness of managing the patient’s health condition.

[...] *there could be shorter intervals.* (P11)

*I always prefer in-person care.* (P21)

## Discussion

This study aimed to understand the perceptions of individuals with T1DM regarding the quality of care provided through the patient navigation program, with a view to improving care for individuals with this condition. The two categories related to care quality that emerged from data analysis were: diabetes management and remote and in-person health services.

In diabetes management, participants reported satisfaction with the care provided by nurses, reflected in expressions such as: “I really liked the care” and “great service.” This positive perception aligns with findings from another study that analyzed patients’ understanding of the disease and treatment adherence^(21)^, emphasizing the importance of the relationship established between healthcare professionals and patients for successful care. Furthermore, participants’ desire to maintain continuous contact with nurses suggests that extended professional support may be crucial for sustaining self-care practices. The appreciation for care and participants’ engagement in the research indicate not only the effectiveness of employed strategies but also the relevance of providing continuous and accessible follow-up in the context of healthcare satisfaction and continuity^(21)^.

Regarding the knowledge gained during consultations, participants’ statements highlight the importance of received guidance, especially on topics not widely covered in conventional medical consultations. This type of health education, focused on practical aspects of self-care, is crucial for improving this aspect in people with DM^(8,10)^. Participants’ expressed satisfaction in receiving detailed information about vaccines and other self-care practices suggests that nurses’ guidance filled significant knowledge gaps, corroborated by studies emphasizing the relevance of educational interventions in improving chronic condition management and promoting health^(8-9)^.

Thus, the improvement in self-care practices reported by study participants, such as foot hydration, are important points in T1DM self-management. Unfortunately, in Brazil, a study conducted in the southeast region showed that interviewees with T1DM and Type 2 Diabetes Mellitus (T2DM) did not perform all foot self-care measures considered essential, such as daily inspection, hydration, nail trimming, drying between toes, and wearing appropriate footwear. Similarly, it was observed that there was an association between lower education levels and reduced ability to perform foot self-care measures^(22)^. According to a study conducted in India with the same population, most diabetic foot complications can be avoided with foot care education, regular foot assessments as a standard of care, and self-care as a preventive strategy^(23)^.

It is important to highlight that patients’ understanding of self-care is essential for successful disease control and, undeniably, for preventing chronic diabetes complications. It is assumed that self-care — when based on diabetes education and regularly followed up with shorter intervals—can foster a trust bond between patients and healthcare teams while also increasing individuals’ willingness to engage in self-care. A cross-sectional study with T1DM patients that assessed the association between diabetes knowledge and self-care through questionnaires showed that participants with greater disease knowledge had higher adherence to self-care^(24)^.

In remote and in-person health services — especially remote — participants highlighted the availability of health services to assist with DM self-management during the COVID-19 pandemic. In China, individuals with T1DM who received remote follow-up during the same period showed reductions in fasting blood glucose levels and glycated hemoglobin (HbA1c) compared to those followed through conventional outpatient consultations^(25)^. Similarly, diabetes self-management was evaluated in a study conducted in Pakistan during the COVID-19 pandemic with 203 patients. The study employed a support, appreciation, learning, and skill transfer method and showed better behavioral practices and self-care among patients in the intervention group^(26)^. Despite the challenges that COVID-19 brought to diabetes treatment, remote care expanded from 1% to 70% in some health centers during this period, enabling continued assistance and improving glycemic control, self-care, and diabetes knowledge^(10,27-28)^.

Additionally, regarding participants’ desire for continued access to services provided during the study, we believe it is feasible to implement patient navigation for T1DM at the institution since it is possible to reorganize existing human resources. Moreover, this care model already exists in other patient assistance programs and could be adapted for this population. Diabetes patients who received personalized follow-up — including patient navigation — improved their follow-up rates with healthcare teams up to fourfold, underwent more frequent exams, showed better glycemic control, and improved their knowledge about the disease and self-care^(8,10,29)^.

Although we experienced significant technological advances during the COVID-19 pandemic, healthcare practices in Brazil remain a major challenge for current care and management models. Knowledge about information technologies, communication, and new care models — both among patients and healthcare teams — contributes to planning humanized care strategies. Recognizing and understanding T1DM patients’ perceptions of patient navigation during COVID-19 is an important first step toward developing clinical awareness of this care modality.

Although this study has valuable findings on T1DM patients’ perceptions of patient navigation during COVID-19, like any research, there are limitations. These include the fact that the study population came from only one tertiary care center that is a reference for this type of care, with patients from across the state. Another limitation is not returning transcribed interview texts to participants, a condition that may have affected results given the methodological rigor adopted. Although phone interviews may have prevented researchers from capturing certain data, such as interviewees’ non-verbal language, they allowed access to participants during social isolation and deepened knowledge on the subject at a time when in-person research would not have been possible. Moreover, no previous Brazilian study has explored T1DM patients’ perceptions of patient navigation during COVID-19, a unique situation that may recur and for which we must be prepared.

Additionally, it is worth noting that nursing’s holistic approach prepares nurses to act as a link between patients, families, and healthcare teams, aligned with patient navigation objectives^(6)^. In other words, nurses are well-positioned to work in various sectors and provide appropriate, efficient, and quality care. Therefore, this study has significant implications for advancing scientific knowledge in health and nursing as it reflects the perceptions of those receiving navigation. Thus, it contributes to a still underexplored field of knowledge and offers insights for future research and practices in this area. Investing in research in this field not only improves clinical practices but also strengthens nursing’s role as an essential pillar for promoting health and well-being among people with DM.

## Conclusion

Based on these results and after analyzing participants’ perceptions in this study, it was possible to understand that the quality of care offered by the patient navigation program is generally positive. Patient navigation benefited individuals with T1DM in their self-care practices, so much so that users requested continuity of this care model, seeing it as an opportunity beyond the difficulties and limitations imposed by treatment. Furthermore, its remote implementation enabled individualized and high-quality care during a period when in-person services were unavailable.

This study also identified important perceptions from individuals with T1DM regarding patient navigation during COVID-19. The experiences and knowledge generated during a crisis are fundamental for developing more effective and humanized care strategies that consider both patient needs and new technological and organizational realities.

Finally, this study provides new insights into research on patient navigation for non-communicable chronic diseases. Given its focus on T1DM patients’ perceptions—who received this care and expressed their views—it also considers the mutual relationship between health conditions and contextual factors that interfere with care provided to these individuals in Brazil.
