# Supplementary material for: User perception of quality of care in patient navigation for type 1 diabetes mellitus
Source: Rev Lat Am Enfermagem. 2025 Mar 14;33:e4491. doi: 10.1590/1518-8345.7350.4491 (PMC11912819; doi:10.1590/1518-8345.7350.4491)
Supplement: Translation

# Translation: Percepção dos usuários sobre qualidade do atendimento na navegação de pacientes com diabetes tipo 1 *

**DOI:** 10.1590/1518-8345.7350.4492

**Authors:** Luciana Foppa, Betina Nemetz, Rosimeri de Matos, Beatriz D’Agord Schaan

**Affiliations:** 1 Hospital de Clínicas de Porto Alegre, Serviço de Enfermagem Ambulatorial, Porto Alegre, RS, Brasil; 2 Universidade Federal do Rio Grande do Sul, Escola de Enfermagem, Porto Alegre, RS, Brasil; 3 Hospital São Lucas da Pontifícia Universidade Católica do Rio Grande do Sul, Porto Alegre, RS, Brasil; 4 Universidade Federal do Rio Grande do Sul, Porto Alegre, RS, Brasil

**Keywords:** Navegação de Pacientes, Diabetes Mellitus Tipo 1, Qualidade da Assistência à Saúde, Cuidados de Enfermagem, Assistência Centrada no Paciente, Consulta Remota

## Abstract

compreender a percepção, das pessoas com diabetes mellitus tipo 1, quanto à qualidade do atendimento por programa de navegação de pacientes.

estudo descritivo com abordagem qualitativa, realizado em hospital universitário na região Sul do Brasil. Participaram 35 pessoas com diabetes mellitus tipo 1. Para coleta de dados, utilizaram-se entrevistas semiestruturadas por teleconsulta. Os dados foram submetidos à análise de conteúdo temática.

dos 35 (100%) participantes, 18 (51,4%) eram homens, com média de idade de 45 (±13) anos. Duas categorias temáticas e três principais temas sobre a qualidade do atendimento emergiram da análise dos dados: gerenciamento do diabetes (qualidade do atendimento em saúde prestado; conhecimento adquirido durante as consultas; melhoria nas práticas de autocuidado como resultado dos cuidados de saúde recebidos) e serviços de saúde remotos e presenciais (disponibilidade dos serviços de saúde durante a pandemia; desejo de acesso continuado aos serviços disponibilizados pelas enfermeiras; importância dos atendimentos recebidos).

após análise das percepções dos participantes, foi possível compreender que a qualidade do atendimento oferecido pelo programa de navegação de pacientes é, em geral, positiva. Tanto que os usuários solicitaram continuidade deste modelo de assistência, vislumbrando uma oportunidade para além das dificuldades e das limitações impostas pela terapêutica.

## Destaques

**(1)** A qualidade do atendimento pelo programa de navegação de pacientes foi positiva. **(2)** A navegação de pacientes proporciona novos rumos para pesquisas em doenças crônicas. **(3)** A navegação de pacientes remota oportunizou um cuidado individualizado e de qualidade. **(4)** Práticas de autocuidado no DM1 podem ser estimuladas com a navegação de pacientes.

## Introdução

Em 2022, havia 8,75 milhões de pessoas vivendo com diabetes mellitus tipo 1 (DM1) em todo o mundo, sendo o Brasil o terceiro país com maior número de casos em todas as idades^(1-2)^. O DM1 é uma condição que requer atenção constante no seu manejo, envolve administração diária de insulina, monitoramento regular da glicemia capilar, adoção de um estilo de vida saudável e prática regular de exercícios físicos^(3)^. Essas atividades demandam do paciente e de sua família um papel central, que envolve educação em diabetes por parte da equipe multidisciplinar, além de apoio emocional e adesão ao tratamento contínuo^(4-5)^. Para tanto, os sistemas de saúde precisam dar suporte, preparar e apoiar os indivíduos acometidos pela doença e os profissionais responsáveis por seu tratamento. A navegação de pacientes é um programa personalizado para atender às necessidades individuais, ajudando continuamente os pacientes com doenças crônicas que demandam autocuidado a superar barreiras modificáveis ao atendimento e atingir seus objetivos^(6)^.

A navegação de pacientes surgiu no final da década de 1980, nos Estados Unidos, com a finalidade de melhorar a adesão ao tratamento e superar barreiras que dificultam o acesso aos serviços de saúde em oncologia. Os resultados deste programa trouxeram benefícios na educação e no acesso ao diagnóstico e tratamento precoce, assim como aumento na taxa de sobrevida de pacientes com câncer^(7)^. Atualmente os programas de navegação de pacientes estão cada vez mais em uso por pessoas com doenças crônicas não transmissíveis em todo o mundo, com resultados positivos em sua maioria^(6)^. Estudos internacionais sobre programa de navegação de pacientes em diabetes têm investigado como esse modelo de cuidado pode melhorar o gerenciamento da doença, a qualidade de vida dos pacientes e os resultados em saúde^(8-10)^. Entretanto, no Brasil, a navegação de pacientes para pessoas com doenças crônicas ainda está em desenvolvimento, existindo poucos estudos publicados sobre o tema^(10)^.

O Conselho Federal de Enfermagem normatizou a atuação do enfermeiro navegador, por meio da Resolução COFEn nº 735/2024. Esta representa um avanço significativo na enfermagem brasileira, ao reconhecer o enfermeiro navegador como um elemento central no cuidado integrado e coordenado de pacientes^(11)^. Inspirada por modelos internacionais, mas adaptada às realidades brasileiras, a navegação de pacientes tem se mostrado uma estratégia promissora para superar barreiras no acesso à saúde e melhorar os resultados clínicos^(9,12)^.

O diabetes mellitus (DM) é um importante fator de risco para doenças cardiovasculares, doença renal crônica, amputação de membros inferiores e infecções, mais recentemente, foi incluída a COVID-19 grave dentre estas. Vale ressaltar que o mau controle glicêmico está associado a um alto risco de agravamento desta infecção, com maior chance de desenvolvimento de síndrome respiratória aguda grave, internação em unidade de tratamento intensivo e morte^(13-14)^. São necessários comportamentos de autogestão do diabetes para a prevenção destas complicações, assim, buscando bom controle glicêmico para reduzir o risco de complicações e melhorar os resultados de saúde^(15)^. Portanto, uma assistência centrada no paciente é importante para auxiliar as pessoas com diabetes a desenvolverem habilidades de autogerenciamento que são necessárias para conviver com sua doença.

Destaca-se que programas de navegadores de pacientes são conduzidos por profissionais com conhecimento e habilidades na área, em sua maioria enfermeiros que prestam assistência centrada no paciente, sendo assim, ajudando a superar barreiras modificáveis ao atendimento e a atingir seus objetivos de assistência, fornecendo uma abordagem personalizada para atender às necessidades individuais^(6,8)^. Dependendo das barreiras visadas, as tarefas específicas dos navegadores podem incluir um ou mais dos seguintes: educação sobre doenças, educação sobre o sistema de saúde, remoção de barreiras entre médico e paciente, abordagem de outras barreiras financeiras, auxílio na coordenação de cuidados, orientação sobre recursos comunitários, entre outros^(6)^.

Logo, estudos têm sido realizados para investigar quais barreiras de autocuidado relacionadas ao diabetes os pacientes vêm relatando^(16-17)^. Entre os obstáculos à autogestão da diabetes que foram citados pelos pacientes está: a lacuna de comunicação entre usuários e profissionais de saúde, a má compreensão da condição da doença, bem como as dificuldades financeiras^(16-17)^. Apesar dos avanços no cuidado à saúde e das iniciativas para melhorar a assistência de pessoas com doenças crônicas, como o DM, existe uma lacuna significativa no conhecimento e na implementação de programas de navegação de pacientes no Brasil. Essa lacuna ressalta a necessidade de mais pesquisas sobre a navegação de pacientes no país em questão. Compreender como programas de navegação de pacientes podem ser adaptados e implementados no contexto brasileiro é essencial para melhorar o cuidado das pessoas com DM, contribuindo para a redução de desigualdades em saúde e a melhoria da qualidade do atendimento. Diante de tais circunstâncias, destaca-se a importância de compreender a percepção das pessoas com DM1 quanto à qualidade do atendimento no programa de navegação de pacientes, com o intuito de aperfeiçoar o planejamento de ações e intervenções para pessoas com esta condição, por meio de uma abordagem integrada e centrada no paciente, que leve em consideração a complexidade da doença e as necessidades individuais de cada paciente.

Considerando os objetivos da navegação de pacientes e a complexidade das pessoas com DM1 atendidos na atenção terciária, identifica-se a necessidade de realizar estudos que versem sobre a atuação do enfermeiro neste programa. Desta forma, o objetivo deste estudo foi compreender a percepção das pessoas com DM1 quanto à qualidade do atendimento por programa de navegação de pacientes.

## Método

### Tipo e local do estudo

Trata-se de um estudo descritivo, com abordagem qualitativa, por meio de análise de conteúdo temática de Bardin^(18)^, realizado em um hospital universitário do Sul do Brasil. Neste estudo, seguiram-se as recomendações do *Consolidated Criteria for Reporting Qualitative Studies* (COREQ)^(19)^.

No hospital em questão, foram realizadas, em 2023, em torno de 547.805 consultas ambulatoriais. Além disso, foram realizadas mais de 31 mil teleconsultas^(20)^. O ambulatório de endocrinologia da instituição, campo da pesquisa, conta com o atendimento de médicos endocrinologistas, enfermeiros, assistente social e nutricionistas. Esse ambulatório é referência para tratamento de pessoas com DM1 de diversas idades, também são atendidos pacientes provenientes de todas as regiões do estado do Rio Grande do Sul.

### População e participantes

A população foi composta por pacientes com diagnóstico de DM1, com acompanhamento regular no ambulatório de endocrinologia da instituição. Os participantes foram selecionados de forma intencional e eram pessoas provenientes do estudo da tese de doutorado, intitulada “Autogestão do diabetes tipo 1 em adultos: uma abordagem centrada na pessoa para melhorar o conhecimento e a adesão ao tratamento em hospital terciário”, junto ao Programa de Pós-Graduação em Endocrinologia da Universidade Federal do Rio Grande do Sul.

Para inclusão no estudo, os participantes deveriam ter mais de 18 anos e diagnóstico de DM1. Os critérios de exclusão foram: déficit cognitivo, gravidez, hipoacusia e dependência química. O critério de saturação dos dados, isto é, a repetição de dados nas entrevistas foi utilizado para definir o quantitativo de participantes. Os pacientes foram selecionados progressivamente para integrar o estudo, até o momento em que a saturação dos dados foi atingida, resultando em 35 participantes. A saturação de dados ocorreu pois a coleta adicional de informações não resultou em novas categorias ou *insights* relevantes, indicando que o conhecimento necessário para responder à questão de pesquisa foi plenamente explorado^(18)^.

### Coleta de dados

A coleta de dados foi realizada por uma única pesquisadora, mestre em enfermagem, durante seu doutorado. A pesquisadora possui experiência em educação em diabetes. Cabe ressaltar que a tese de doutorado abordou, como estratégia para melhorar a autogestão do diabetes, a navegação de pacientes. Além do mais, envolveu na intervenção três enfermeiras e uma acadêmica de enfermagem. Para evitar viés nas entrevistas, os participantes que foram acompanhados pelas outras duas enfermeiras e pela acadêmica de enfermagem durante o desenvolvimento do estudo principal, foram entrevistados pela pesquisadora para compor este estudo, ou seja, os pacientes não tinham vínculo com a entrevistadora. Outro dado importante a destacar é que a navegação de pacientes foi desenvolvida durante a pandemia de COVID-19, de janeiro de 2021 a abril de 2022.

Ademais, as informações foram coletadas por meio de entrevistas semiestruturadas, nas quais foram abordadas questões abertas sobre o atendimento oferecido pelas enfermeiras navegadoras durante a pesquisa, o uso de mensagens de texto e o atendimento por teleconsulta e presencial oferecido aos pacientes com DM1 da instituição. Logo, foram realizadas as seguintes perguntas: Como o atendimento recebido influenciou suas práticas de autocuidado? Como você avalia a disponibilidade dos serviços (teleconsulta, mensagens de texto e atendimento presencial) que você recebeu? Você tem alguma sugestão de melhoria para o atendimento recebido? Além do mais, as entrevistas ocorreram por telefone e em horário indicado pelo participante, com tempo médio de duração de 21 minutos, 30 dias após o término do atendimento com a enfermeira navegadora, ocorrido entre os meses de janeiro e abril de 2022. As ligações foram realizadas em uma sala destinada a teleconsultas, na instituição campo da pesquisa.

Por fim, os depoimentos dos pacientes foram gravados por meio de um aplicativo de ligações disponibilizado no computador da instituição. Já as notas de campo foram realizadas pela pesquisadora durante as entrevistas, com auxílio de formulário *online* criado para esse fim. Cumpre esclarecer que não houve validação das falas que integraram o corpo do relatório da pesquisa. O estudo incluiu um projeto-piloto em caráter experimental, por duas vezes, para averiguação da adequação da linguagem utilizada e da compreensão das questões.

### Análise de dados

Na medida em que as entrevistas se encerravam, eram transcritas literalmente e confrontadas com as informações registradas durante as interlocuções telefônicas. As transcrições foram revisadas, conferindo o registro no formulário *online* (notas de campo) com o áudio gravado. Os dados sociodemográficos dos participantes foram registrados no programa *Microsoft Excel*.

Para garantir o anonimato e respeitar os princípios éticos, as entrevistas foram codificadas pelas siglas: P1, P2, P3…, cuja numeração crescente correspondeu ao ordenamento cronológico das entrevistas. Da mesma forma, o uso das informações obtidas foi apenas para fins deste estudo.

A partir das transcrições na íntegra das entrevistas e a fim de manter o rigor metodológico, as informações foram submetidas à análise de conteúdo temática de Bardin, realizada em três etapas, que consistem em: pré-análise (transcrição, constituição do corpus, leitura flutuante das entrevistas e formulação de hipóteses e objetivos), exploração do material (exportação do material para o *software NVivo*® versão 15, classificação dos dados e incorporação das informações, codificação e categorização temática) e tratamento dos resultados obtidos e interpretação (articulações das inferências e interpretação dos dados, bem como retomada do referencial teórico)^(18)^. Além disso, a aplicação de critérios de qualidade que envolvem a exaustividade, representatividade, pertinência e objetividade^(18)^ foram valiosos para refletir com precisão as experiências e as realidades dos participantes. Da mesma forma que, como parte do processo de análise, a percepção dos participantes foi entendida conforme a maneira como os entrevistados expressavam suas vivências e experiências, além de como essas expressões são interpretadas e categorizadas durante a análise^(18)^.

Ademais, a interpretação dos dados foi realizada à luz de fundamentação teórica, que se alicerçou no efeito de navegação do paciente sobre o autocuidado, conhecimento sobre a doença e acompanhamento com a equipe de saúde^(7,10)^. O processo de análise dos dados consistiu na organização destes por meio da identificação, codificação e criação de categorias. A codificação foi realizada após a leitura e a marcação na transcrição das entrevistas, destacando palavras, frases ou temas pertinentes ao assunto estudado. As categorias foram formadas a partir do agrupamento de códigos ou de expressões que apresentavam características semelhantes, estabelecendo uma relação de complementaridade entre os dados.

Em seguida, decorreu-se a classificação dos dados obtidos com o propósito de alcançar compreensão do texto, recortando-o em categorias temáticas emergentes, por meio da organização das expressões ou das palavras significativas. Posteriormente, transcorreu a classificação e a incorporação das informações que geraram os principais temas apresentados nos resultados. Houve uma releitura cuidadosa das categorias que sumarizam o conteúdo das falas dos entrevistados, exigindo um trabalho intelectual aprofundado.

### Aspectos éticos

O estudo foi aprovado pelo comitê de ética da instituição, via Plataforma Brasil, sob número de CAAE 20380919800005327, contemplando-se as prerrogativas anunciadas na Resolução 466/2012 do Conselho Nacional de Saúde. A pesquisadora seguiu o roteiro de ligação telefônica para convite de pesquisas da instituição, no qual constam três opções para o participante enviar o Termo de Consentimento Livre e Esclarecido (*e-mail*, *WhatsApp* ou mensagem), e o documento foi enviado conforme preferência.

## Resultados

A caracterização sociodemográfica demonstrou que dos 35 participantes, 18 (51,4%) eram homens, a idade média era de 45 (±13) anos e 4 (11,4%) eram tabagistas ativos. Os demais dados sociodemográficos estão representados na Tabela 1.

**Tabela 1 -:** Características sociodemográficas e clínicas dos participantes do estudo (n = 35). Porto Alegre, RS, Brasil, 2022

Característica*	**n= 35**
Idade (anos)	45 ± 13
Sexo	18 (51,4)
Masculino	17 (48,6)
Feminino	
Estado civil	23 (65,7)
Casados (as) ou companheiro (a) fixo (a)	9 (25,7)
Solteiros (as)	3 (8,6)
Viúvos (as)	
Tabagismo ativo	4 (11,4)
Ocupação profissional	19 (54,2)
Atividade remunerada	3 (8,6)
Desempregados	13 (37,2)
Aposentados	
Tempo de diagnóstico	26,9 ±11,4
Hemoglobina glicada (%)	8,0 ±1,2
Comorbidades ^†^	9 (25,7)
Hipertensão arterial sistêmica	6 (17,1)
Doenças psiquiátricas	6 (17,1)
Dislipidemia	19 (54,2)
Retinopatia	3 (8,6)
Doença renal do diabetes	6 (17,1)
Neuropatia sensitiva ^‡^	2 (5,7)
Lesões nos pés	
Escolaridade	3 (8,6)
Ensino fundamental	18 (51,4)
Ensino médio	14 (40)
Ensino superior	
Insulina pré-prandial	6 (17,1)
Regular	29 (82,9)
Análogos	
Insulina basal	15 (42,9)
Protamina Neutra de Hagedorn (NPH)	20 (57,1)
Análogos	

*Variáveis contínuas descritas por média e desvio-padrão e categóricas por número absoluto e percentil; ^†^Foi computada mais de uma resposta a esta variável; ^‡^Neuropatia sensitiva foi considerada presente quando registrado em prontuário pelo médico assistente

Na Figura 1, apresentam-se as categorias temáticas e os principais temas oriundos do processo de pré-análise e análise de dados que emergiram das falas dos participantes.

**Figura 1 -:** Categorias temáticas e principais temas resultantes de análise das falas dos participantes (n = 35). Porto Alegre, RS, Brasil, 2022

### Gerenciamento do diabetes

Nesta categoria foram agrupadas percepções dos participantes acerca da qualidade do atendimento prestado, do conhecimento adquirido durante as consultas e da melhoria nas práticas de autocuidado como resultado dos cuidados de saúde recebidos.

Quanto à qualidade do atendimento prestado, os participantes relataram satisfação relacionada ao serviço. As entrevistas que salientaram este tema foram: P1, P2, P3, P4, P7, P11, P15, P16, P17, P20, P29, P30, P34 e P35. Em sua maioria, os participantes gostaram da assistência oferecida e almejavam que fosse mantido o acompanhamento com a navegação de pacientes na instituição. A qualidade percebida pode estar fortemente associada às interações com profissionais específicos, como enfermeiras que desempenham um papel central no cuidado. Destacam-se as falas a seguir:

*Parabéns pela pesquisa, gostei muito! Gostei muito do atendimento da enfermeira.* (P2)

*Atendimento muito bom! Gostaria de continuar tendo algum contato para recorrer em caso de dúvidas.* (P7)

*Gostei muito de participar da pesquisa! [...], atendimento ótimo [...].* (P11)

As entrevistas que expressaram opinião sobre o conhecimento adquirido durante as consultas foram: P1, P5, P6, P10, P14, P16, P18, P20, P22, P25, P26, P28 e P32. A educação em saúde desempenha um papel importante na capacitação dos pacientes para o autocuidado, a prevenção de complicações e a adesão ao tratamento. Alguns entrevistados referiram que aprenderam novas rotinas de cuidados com os pés durante as consultas, já outros aludiram sobre as orientações fornecidas em relação às vacinas e ao uso da insulina. Ressaltam-se os trechos a seguir:

[...] *gostei muito de receber as orientações, principalmente sobre as vacinas.* (P1)

[...] *ajudou com questões que nunca tinha sido orientada pelos médicos a respeito da aplicação da insulina.* (P14)

*Gostei de aprender como hidratar os pés e cotovelos.* (P18)

As entrevistas que mencionaram a melhoria nas práticas de autocuidado como resultado dos cuidados de saúde recebidos foram: P9, P11, P12, P13, P18, P24, P25, P26, P27, P28 e P31. A navegação de pacientes pode ser estruturada não apenas para fornecer informações, mas também para preparar os pacientes a superar obstáculos e incorporar mudanças de forma sustentável. Todas essas entrevistas referiram como a navegação de pacientes auxiliou os entrevistados a aprender mais sobre o diabetes e sua gestão. Essas conclusões surgiram nas falas a seguir:

*Achei muito bom! Bem esclarecedora a pesquisa, passei a realizar cuidados que não fazia antes, acho que talvez por desconhecimento* [...]. (P9)

[...] *muito válido lembrar das coisas que caem no esquecimento* [...]. (P11)

*Gostei de aprender novas rotinas, melhorei demais os cuidados.* (P18)

[...] *tenho diabetes há 31 anos e sempre estamos aprendendo alguma coisa.* (P31)

Tendo em vista que as entrevistas destacaram que a navegação de pacientes contribuiu significativamente para o aprimoramento das práticas de autocuidado, especialmente na gestão do diabetes. É fundamental examinar como a disponibilidade e o formato dos serviços de saúde, seja remoto ou presencial, afetam essa interação e a continuidade do cuidado.

### Serviços de saúde remotos e presenciais

Nesta categoria foram agrupadas percepções dos participantes acerca da disponibilidade dos serviços de saúde durante a pandemia, bem como o desejo de acesso continuado aos serviços disponibilizados pelas enfermeiras e a importância dos atendimentos recebidos.

As entrevistas que tinham informações a respeito da disponibilidade dos serviços de saúde para auxiliar o autogerenciamento do diabetes foram: P5, P8, P11, P15, P16, P19, P20, P24 e P35. Estes pacientes referiram gratidão pela viabilidade dos atendimentos que receberam durante a pandemia de COVID-19 e com as medidas de isolamento social que a acompanharam. A pandemia trouxe desafios sem precedentes para os sistemas de saúde, afetando tanto a oferta quanto a demanda por serviços, particularmente para pessoas com DM, que requerem acompanhamento contínuo. Ressaltam-se as falas a seguir:

[...] *quero agradecer, gostei do atendimento durante a pandemia.* (P19)

*Achei muito boa a pesquisa, deixa a gente mais seguro, passa segurança. Agradeço a disponibilidade de ligar à noite, porque trabalho durante o dia.* (P24)

*Agradecer o contato e dizer que foi muito importante o cuidado de vocês, principalmente durante a pandemia, pois foi uma forma de acompanhamento no período de isolamento.* (P35)

Os participantes relataram, também, o desejo de acesso continuado aos serviços disponibilizados pelas enfermeiras após o término da pesquisa e de forma remota. As entrevistas que destacaram informações a respeito do tema foram: P2, P3, P6, P8, P11, P20, P24, P29 e P33, e as falas das quais emergiram o interesse em continuar com a navegação de pacientes foram: P3, P4, P7, P15, P20, P23, P29, P32 e P34. A demanda dos participantes pela continuidade dos serviços remotos aponta para uma possível lacuna nos sistemas tradicionais de saúde. Essas informações estão contidas nas falas a seguir:

*Deveria continuar o atendimento por telefone e essa forma de atenção diferenciada que vocês dedicam, se pudesse continuar com o* WhatsApp *seria melhor.* (P3)

*Gostaria que continuasse, pois ajudou muito no meu cuidado, foi muito útil, é muito bom ter alguém com quem conversar.* (P4)

*Gostaria que mantivesse. As consultas presenciais são muito espaçadas e esse atendimento remoto traz mais uma forma de um compromisso, que vai ser atendido e tem que fazer as coisas certas. Gostei muito do* online*! Gostei de avaliar os pés!* (P20)

[...] *acho essencial ter telefone de contato* [...]. (P23)

As entrevistas que trouxeram informações sobre a importância dos atendimentos recebidos foram: P1, P2, P5, P7, P9, P12, P13, P15, P16, P17, P22, P23, P24, P25 e P34. Estas trataram sobre a percepção de ter uma pessoa para contato nas horas de dúvidas e o quanto aprenderam com a educação em saúde oferecida pelas enfermeiras durante a navegação de pacientes. Em contextos de atendimento à saúde, especialmente para DM, o relacionamento contínuo e o suporte oferecido pelos profissionais de saúde podem ser fatores determinantes para o sucesso do tratamento e para o bem-estar do paciente. Destacam-se as falas a seguir:

*Achei ótimo as consultas, aprendi bastante* [...]. (P5)

[...] *adorei e foi muito boa a experiência; me senti mais acolhida* [...]*.* (P15)

[...] *aprendi muito na pesquisa, fiquei sabendo coisas que os médicos não falam* [...]. (P25)

[...] *muito agradecido pelo atendimento, atenção e pela educação na qual sempre tratou as conversas e exames.* (P34)

No entanto, houve um entrevistado que relatou descontentamento quanto aos atendimentos remotos e outro quanto ao intervalo entre as consultas. Embora o atendimento remoto tenha surgido como uma solução viável para manter o acompanhamento de pacientes em tempos de distanciamento social, o mesmo pode não atender igualmente a todos. Além disso, o intervalo entre as consultas pode influenciar não apenas a satisfação com o serviço, mas também a eficácia do manejo da condição de saúde do paciente.

*[...] poderia ter intervalo mais curto.* (P11)

*Prefiro sempre atendimento presencial.* (P21)

## Discussão

Este estudo buscou compreender as percepções de pessoas com DM1 quanto à qualidade do atendimento e por meio do programa de navegação de pacientes, com vistas à melhoria do atendimento aos indivíduos com esta condição. As duas categorias sobre a qualidade do atendimento que emergiram da análise dos dados foram: gerenciamento do DM e serviços de saúde remotos e presenciais.

No gerenciamento do DM, os participantes relataram satisfação quanto ao atendimento prestado pelas enfermeiras, refletido pelas expressões como: “gostei muito do atendimento” e “atendimento ótimo”. Essa percepção positiva corrobora com os achados de outro estudo que analisou a assimilação de pacientes sobre a doença e a adesão ao tratamento^(21)^, que destaca a importância do vínculo estabelecido entre o profissional de saúde e o paciente para o sucesso do cuidado. Além disso, o desejo dos participantes de manter um contato contínuo com as enfermeiras sugere que o suporte profissional estendido pode ser crucial para a manutenção de práticas de autocuidado. A valorização do atendimento e o engajamento dos participantes na pesquisa indicam não apenas a eficácia das estratégias empregadas, mas também a relevância de proporcionar um acompanhamento contínuo e acessível, no contexto da satisfação e continuidade dos cuidados em saúde^(21)^.

Quanto ao conhecimento adquirido durante as consultas, as falas dos participantes destacam a importância das orientações recebidas, especialmente com assuntos não amplamente abordados durante as consultas médicas convencionais. Esse tipo de educação em saúde, focada em aspectos práticos do autocuidado, é crucial para melhorar este aspecto em pessoas com DM^(8,10)^. A satisfação expressa pelos participantes, ao receberem informações detalhadas sobre vacinas e outras práticas de autocuidado, sugere que as orientações fornecidas pelas enfermeiras preencheram lacunas significativas no conhecimento dos pacientes, o que é corroborado por estudos que ressaltam a relevância de intervenções educativas na melhoria do manejo de condições crônicas e na promoção da saúde^(8-9)^.

Logo, a melhoria nas práticas de autocuidado relatada pelos participantes do estudo, como exemplo a hidratação dos pés, é um ponto importante na autogestão do DM1. Infelizmente, no Brasil, estudo realizado na região sudeste evidenciou que os entrevistados com diabetes (tipo 1 e 2) não realizavam todas as medidas consideradas de autocuidado com os pés, como inspeção diária, hidratação, corte das unhas, secagem dos espaços interdigitais e uso de calçado adequado. Similarmente, observou-se que havia associação entre a menor escolaridade e a menor capacidade de realização de medidas de autocuidado com os pés^(22)^. Segundo estudo desenvolvido na Índia com a mesma população, a maioria das complicações do pé diabético podem ser evitadas com educação sobre os cuidados com os pés, avaliações regulares dos pés como padrão de tratamento e autocuidado como estratégia preventiva^(23)^.

É relevante destacar que a compreensão do paciente sobre o autocuidado é essencial para obter êxito no controle de sua doença e, incontestavelmente, na prevenção das complicações crônicas do diabetes. Pressupõe-se que o autocuidado - quando alicerçado na educação em diabetes e acompanhado regularmente, com intervalos mais curtos - pode favorecer o desenvolvimento de um vínculo de confiança entre paciente e equipe de saúde, além de poder intensificar a disposição da pessoa com diabetes em realizar o autocuidado. Estudo transversal com pacientes com DM1, que avaliou a associação entre o conhecimento sobre diabetes e o autocuidado por meio de questionários, demonstrou que os participantes com maior conhecimento sobre a doença apresentaram maior adesão ao autocuidado^(24)^.

Nos serviços de saúde remotos e presenciais, mas principalmente no remoto, foi destacada pelos entrevistados a disponibilidade dos serviços de saúde para auxiliar o autogerenciamento do DM durante a pandemia de COVID-19. Pessoas com DM1, na China, que receberam acompanhamento remoto no mesmo período, apresentaram redução nos níveis de glicemia em jejum e hemoglobina glicada (HbA1c), isso quando comparados aos acompanhados por consulta ambulatorial convencional^(25)^. Da mesma forma, o autogerenciamento do diabetes foi avaliado no estudo realizado no Paquistão durante a pandemia de COVID-19, em 203 pacientes, e que empregou o método de apoio, apreciação, aprendizagem e transferência de competências, além de apresentar melhores práticas comportamentais e de autocuidado para os pacientes do grupo intervenção^(26)^. Apesar de dificuldades que a pandemia de COVID-19 trouxe para o tratamento do diabetes, a expansão do atendimento remoto neste período passou de 1% para 70%, em alguns centros de saúde, possibilitando a continuidade na assistência e melhorando o controle glicêmico, o autocuidado e o conhecimento sobre o diabetes^(10,27-28)^.

Ainda, nos serviços de saúde remotos e presenciais, quanto ao desejo de acesso continuado aos serviços disponibilizados durante o estudo relatado pelos participantes, acreditamos que seja viável a implantação da navegação de pacientes com DM1 na instituição, pois é possível reorganizar recursos humanos já existentes na instituição. Além do mais, este modelo de cuidado já existe em outros programas de assistência ao paciente, podendo ser adaptado a este público. Pacientes com diabetes que receberam acompanhamento personalizado, o que inclui a navegação de pacientes, melhoraram até quatro vezes as taxas de acompanhamento com a equipe de saúde, realizaram exames mais frequentemente, apresentaram melhora no controle glicêmico e melhoraram o conhecimento sobre a doença e seu autocuidado^(8,10,29)^.

Apesar de vivenciarmos importantes avanços tecnológicos durante a pandemia de COVID-19, as práticas de atenção à saúde no cenário brasileiro ainda são um grande desafio aos modelos de atenção e de gestão vigentes. O conhecimento acerca das tecnologias de informação, comunicação e de novos modelos de assistência, tanto por parte dos pacientes quanto da equipe de saúde, contribui para o planejamento de estratégias de assistência humanizada. Reconhecer e compreender a percepção das pessoas com DM1 sobre navegação de pacientes, durante a pandemia de COVID-19, é um importante primeiro passo para o desenvolvimento da conscientização clínica desta modalidade de assistência.

Embora este estudo tenha valiosos achados sobre as percepções das pessoas com DM1 sobre navegação de pacientes durante a pandemia de COVID-19, como em qualquer pesquisa, há limitações. Nas quais incluem a população estudada pertencer a apenas um centro de atendimento terciário, porém referência neste tipo de atendimento e com pacientes oriundos de todo o estado. O fato de não retornar o texto transcrito das entrevistas aos participantes, condição que pode ter afetado os resultados diante do rigor metodológico adotado, é outra limitação deste estudo. Ainda que as entrevistas telefônicas possam ter impedido o pesquisador de capturar certos dados, como a linguagem não verbal dos entrevistados, permitiram chegar às pessoas durante o período de isolamento social e aprofundar o conhecimento do tema em estudo no momento em que a pesquisa presencial não teria sido possível. Além disso, nenhum estudo brasileiro anterior explorou a percepção das pessoas com DM1 sobre navegação de pacientes durante a pandemia de COVID-19, uma situação de exceção que pode vir a se repetir e para a qual devemos estar preparados.

Ademais, cabe destacar que a natureza holística da assistência de enfermagem prepara os enfermeiros para atuar como elo entre pacientes, familiares e equipe de saúde, o que vai ao encontro dos objetivos da navegação de pacientes^(6)^. Ou seja, os enfermeiros estão em uma posição favorável para atuar em vários setores e proporcionar assistência adequada, eficiente e de qualidade. Por isso, este estudo apresenta implicações significativas para o avanço do conhecimento científico na área de saúde e enfermagem, uma vez que retrata a percepção de quem recebe a navegação. Assim, contribui para um campo de conhecimento ainda pouco explorado e oferece subsídios para futuras pesquisas e práticas na área. Investir em pesquisa nessa área não só aprimora as práticas clínicas, mas também fortalece o papel da enfermagem como um pilar essencial para a promoção da saúde e do bem-estar das pessoas com DM.

## Conclusão

Diante dos resultados, após análise das percepções dos participantes deste estudo, foi possível compreender que a qualidade do atendimento oferecido pelo programa de navegação de pacientes é, em geral, positiva. A navegação de pacientes beneficiou as pessoas com DM1 nas práticas de autocuidado, tanto que os usuários solicitaram continuidade deste modelo de assistência, vislumbrando uma oportunidade para além das dificuldades e limitações impostas pela terapêutica. Além do mais, a sua realização remota oportunizou um cuidado individualizado e de qualidade em um período no qual os atendimentos presenciais não estavam ocorrendo.

Este estudo, também, identificou importantes percepções das pessoas com DM1 em relação à navegação de pacientes durante a pandemia de COVID-19. A vivência e o conhecimento gerado em momento de crise são fundamentais para o desenvolvimento de estratégias mais eficazes e humanizadas de cuidado, que levem em conta tanto as necessidades dos pacientes quanto às novas realidades tecnológicas e organizacionais.

Por fim, este estudo proporciona novas informações para o campo de pesquisas sobre a navegação de pacientes com doenças crônicas não transmissíveis, tendo em vista que se concentra nas percepções dos pacientes com DM1, que receberam este atendimento e expressaram suas percepções, além de considerar a mutualidade entre as condições de saúde e os fatores contextuais que interferem na assistência prestada a esses indivíduos no Brasil.

# Translation: Percepción de las personas con diabetes mellitus tipo 1 sobre la calidad de la atención brindada a través de la navegación de pacientes*

**DOI:** 10.1590/1518-8345.7350.4490

**Authors:** Luciana Foppa, Betina Nemetz, Rosimeri de Matos, Beatriz D’Agord Schaan

**Affiliations:** 1 Hospital de Clínicas de Porto Alegre, Serviço de Enfermagem Ambulatorial, Porto Alegre, RS, Brasil; 2 Universidade Federal do Rio Grande do Sul, Escola de Enfermagem, Porto Alegre, RS, Brasil; 3 Hospital São Lucas da Pontifícia Universidade Católica do Rio Grande do Sul, Porto Alegre, RS, Brasil; 4 Universidade Federal do Rio Grande do Sul, Porto Alegre, RS, Brasil

**Keywords:** Navegación de Pacientes, Diabetes Mellitus Tipo 1, Calidad de la Atención de Salud, Atención de Enfermería, Atención Dirigida al Paciente, Consulta Remota

## Abstract

comprender la percepción de las personas con diabetes mellitus tipo 1 sobre la calidad de la atención brindada a través de un programa de navegación de pacientes.

estudio descriptivo con enfoque cualitativo, realizado en un hospital universitario de la región sur de Brasil. Participaron 35 personas con diabetes mellitus tipo 1 para la recolección de datos. Los datos fueron sometidos a análisis de contenido temático.

de los 35 (100%) participantes, 18 (51,4%) eran hombres, con edad media de 45 (±13) años. Del análisis de los datos surgieron dos categorías temáticas y tres temas principales relacionados con la calidad de la atención: manejo de la diabetes (calidad de la atención en salud brindada; conocimientos adquiridos durante las consultas; mejora de las prácticas de autocuidado como resultado de la atención en salud recibida) y servicios remotos y presenciales (disponibilidad de los servicios de salud durante la pandemia; deseo de seguir accediendo a los servicios que brindan las enfermeras; importancia de la atención recibida).

después de analizar las percepciones de los participantes, se comprobó que la calidad de la atención que brinda el programa de navegación de pacientes es, en general, positiva. Tal es así que los usuarios solicitaron que continue este modelo asistencial, y vieron una oportunidad más allá de las dificultades y limitaciones que impone la terapia.

## Destacados

**(1)** La calidad de la atención del programa de navegación de pacientes fue positiva. **(2)** La navegación de pacientes proporciona nuevas direcciones para la investigación de enfermedades crónicas. **(3)** La navegación de pacientes remota brindó atención personalizada y de calidad. **(4)** Las prácticas de autocuidado de la DM1 se pueden fomentar con la navegación de pacientes.

## Introducción

En 2022, había 8,75 millones de personas que viven con Diabetes *Mellitus* Tipo 1 (DM1) en todo el mundo y Brasil es el tercer país con mayor número de casos en todas las edades^(1-2)^. La DM1 es una condición que requiere atención constante para manejarla, implica administración diaria de insulina, control regular de la glucemia capilar, adopción de un estilo de vida saludable y ejercicio físico regular^(3)^. Estas actividades requieren que el paciente y la familia asuman un papel central, eso implica que es necesario que haya educación en diabetes por parte del equipo multidisciplinario, así como apoyo emocional y adherencia al tratamiento continuo^(4-5)^. Para ello, los sistemas de salud deben ayudar, preparar y apoyar a las personas afectadas por la enfermedad y a los profesionales responsables del tratamiento. La navegación de pacientes es un programa personalizado para satisfacer las necesidades individuales, que ayuda continuamente a los pacientes con enfermedades crónicas que requieren autocuidado a superar barreras modificables para la atención y lograr sus objetivos^(6)^.

La navegación de pacientes surgió a finales de los años 80 en Estados Unidos con el objetivo de mejorar la adherencia al tratamiento y superar las barreras que dificultan el acceso a los servicios de salud oncológicos. Como resultado, dicho programa mejoró la educación y el acceso al diagnóstico y tratamiento temprano y produjo un aumento en la tasa de supervivencia de los pacientes con cáncer^(7)^. Actualmente, las personas con enfermedades crónicas no transmisibles en todo el mundo utilizan cada vez más los programas de navegación de pacientes, cuyos resultados son, en general, positivos^(6)^. Hay estudios internacionales sobre programas de orientación para pacientes con diabetes que han investigado cómo este modelo de atención puede mejorar el manejo de la enfermedad, la calidad de vida de los pacientes y los resultados de salud^(8-10)^. Sin embargo, en Brasil, la navegación de pacientes para personas con enfermedades crónicas aún está en desarrollo y hay pocos estudios publicados sobre el tema^(10)^.

El Consejo Federal de Enfermería estandarizó las funciones del enfermero navegador, a través de la Resolución COFEn N° 735/2024. Esto representa un avance significativo en la enfermería brasileña, dado que reconoce al enfermero navegador como elemento central en la atención integrada y coordinada del paciente^(11)^. Inspirada en modelos internacionales, pero adaptada a la realidad brasileña, la navegación de pacientes ha demostrado ser una estrategia prometedora para superar barreras en el acceso a la atención en salud y mejorar los resultados clínicos^(9,12)^.

La diabetes *mellitus* (DM) es un factor de riesgo importante para enfermedades cardiovasculares, enfermedad renal crónica, amputación de miembros inferiores e infecciones, más recientemente se incluyó entre ellas el COVID-19 grave. Cabe mencionar que un mal control glucémico se asocia con un alto riesgo de que se agrave la infección y mayor probabilidad de desarrollar síndrome respiratorio agudo severo, ingreso a unidad de cuidados intensivos y muerte^(13-14)^. Es necesario que el paciente adquiera conductas de autocontrol de la diabetes para prevenir esas complicaciones, si se tiene un buen control glucémico se reduce el riesgo de complicaciones y mejoran los resultados de salud^(15)^. Por lo tanto, la atención centrada en el paciente es importante para ayudar a las personas con diabetes a desarrollar habilidades de autocuidado, que son necesarias para vivir con la enfermedad.

Cabe destacar que los programas de navegación de pacientes son liderados por profesionales con conocimientos y habilidades en el área, generalmente, enfermeros que brindan atención centrada en el paciente y, de esa forma, ayudan a superar barreras modificables para la atención y lograr los objetivos de atención, y brindan un enfoque personalizado para satisfacer las necesidades individuales^(6,8)^. Dependiendo de las barreras a superar, las tareas específicas de los navegadores pueden incluir una o más de las siguientes: brindar educación sobre enfermedades, brindar educación sobre el sistema de salud, eliminar barreras entre el médico y el paciente, abordar otras barreras financieras, ayudar en la coordinación de la atención, brindar orientación sobre los recursos comunitarios, entre otras^(6)^.

Por consiguiente, se han realizado estudios para investigar cuáles son las barreras para el autocuidado de la diabetes que informaron los pacientes^(16-17)^. Entre los obstáculos para el autocontrol de la diabetes citados por los pacientes se encuentran: falta de comunicación entre los usuarios y los profesionales de la salud, mala comprensión del estado de la enfermedad, así como dificultades financieras^(16-17)^. A pesar de los avances en salud y de las iniciativas para mejorar la atención a personas con enfermedades crónicas, como la DM, existe un vacío importante en el conocimiento y la implementación de programas de navegación de pacientes en Brasil. Ese vacío destaca que es necesario que se realicen más investigaciones sobre la navegación de pacientes en ese país.

Comprender cómo se pueden adaptar e implementar los programas de navegación de pacientes en el contexto brasileño es esencial para mejorar la atención de las personas con DM, contribuir a reducir las desigualdades en salud y mejorar la calidad de la atención. Ante dichas circunstancias, cabe destacar que es importante comprender la percepción que tienen las personas con DM1 sobre la calidad de la atención del programa de navegación de pacientes, con el fin de mejorar la planificación de acciones e intervenciones para las personas con esta condición, mediante un enfoque integrado y centrado en el paciente que tenga en cuenta la complejidad de la enfermedad y las necesidades individuales de cada paciente.

Considerando los objetivos de la navegación de pacientes y la complejidad de las personas con DM1 que acuden a la atención terciaria de salud, se considera que es necesario realizar estudios que se centren en el papel del enfermero en este programa. Por lo tanto, el objetivo de este estudio fue comprender la percepción de las personas con DM1 sobre la calidad de la atención brindada a través de un programa de navegación de pacientes.

## Método

### Tipo y lugar del estudio

Se trata de un estudio descriptivo, con abordaje cualitativo, utilizando el análisis de contenido temático de Bardin^(18)^, realizado en un hospital universitario del sur de Brasil. En este estudio se siguieron las recomendaciones de los *Consolidated Criteria for Reporting Qualitative Studies* (COREQ)^(19)^.

En 2023 se realizaron alrededor de 547.805 consultas ambulatorias en el hospital en cuestión. Además, se realizaron más de 31 mil teleconsultas^(20)^. El servicio ambulatorio de endocrinología de la institución, campo de la investigación, cuenta con endocrinólogos, enfermeros, trabajadores sociales y nutricionistas. Ese servicio ambulatorio es referencia para el tratamiento de personas con DM1 de diferentes edades, también brinda atención a pacientes de todas las regiones del estado de Rio Grande do Sul.

### Población y participantes

La población estuvo compuesta por pacientes diagnosticados con DM1, que reciben seguimiento periódico en el servicio ambulatorio de endocrinología de la institución. Los participantes fueron seleccionados intencionalmente y eran personas que formaban parte del estudio de tesis doctoral, titulado “*Autogestão do diabetes tipo 1 em adultos: uma abordagem centrada na pessoa para melhorar o conhecimento e a adesão ao tratamento em hospital terciário*” (Automanejo de la diabetes tipo 1 en adultos: un enfoque centrado en la persona para mejorar el conocimiento y la adherencia al tratamiento en un hospital terciario), realizado junto con el Programa de Postgrado en Endocrinología de la Universidad Federal de Rio Grande do Sul.

Para ser incluidos en el estudio, los participantes debían ser mayores de 18 años y haber sido diagnosticados con DM1. Los criterios de exclusión fueron: deterioro cognitivo, embarazo, hipoacusia y dependencia química. Para definir el número de participantes se utilizó el criterio de saturación de datos, es decir, la repetición de datos en las entrevistas. Los pacientes fueron seleccionados progresivamente para participar del estudio, hasta alcanzar la saturación de datos, en total se incluyeron 35 participantes. La saturación de datos se produjo cuando la recolección adicional de información no dio como resultado nuevas categorías o *insights* relevantes, lo que indica que se exploró a fondo el conocimiento necesario para responder a la pregunta de investigación^(18)^.

### Recolección de datos

La recolección de datos fue realizada por una sola investigadora, máster en enfermería, durante su doctorado. La investigadora tiene experiencia en educación en diabetes. Cabe señalar que la tesis doctoral abordó la navegación de pacientes como estrategia para mejorar el autocontrol de la diabetes. Además, en la intervención participaron tres enfermeras y una estudiante de enfermería. Para evitar sesgos en las entrevistas, los participantes que recibieron seguimiento por parte de las otras dos enfermeras y de la estudiante de enfermería durante el desarrollo del estudio principal, fueron entrevistados por la investigadora para realizar este estudio, es decir, los pacientes no tenían vínculos con la investigadora. Otro dato importante por destacar es que la navegación de pacientes se desarrolló durante la pandemia de COVID-19, desde enero de 2021 hasta abril de 2022.

Por otro lado, la información fue recolectada a través de entrevistas semiestructuradas, en las que se abordaron preguntas abiertas sobre la atención que brindaron las enfermeras navegantes durante la investigación, el uso de mensajes de texto y la teleconsulta y atención presencial que se les brinda a los pacientes con DM1 en la institución. Por lo tanto, se formularon las siguientes preguntas: ¿Cómo influyó la atención recibida en tus prácticas de autocuidado? ¿Cómo evalúas la disponibilidad de los servicios (teleconsulta, mensajes de texto y atención presencial) que recibiste? ¿Tienes alguna sugerencia para mejorar la atención recibida? Las entrevistas se realizaron por teléfono y en el horario indicado por el participante, tuvieron una duración promedio de 21 minutos, fueron realizadas 30 días después de finalizada la consulta con la enfermera navegante, que se desarrolló entre los meses de enero y abril 2022. Las llamadas se realizaron en una sala designada para teleconsultas, en la institución de investigación.

Por último, las declaraciones de los pacientes fueron registradas mediante una aplicación de llamadas disponible en la computadora de la institución. La investigadora tomó las notas de campo durante las entrevistas, con la ayuda de un formulario *on line* creado para tal fin. Cabe aclarar que no se realizó la validación de los enunciados que conformaron el cuerpo del informe de investigación. El estudio incluyó un proyecto piloto con carácter experimental, dos veces, para verificar la adecuación del lenguaje utilizado y la comprensión de las preguntas.

### Análisis de los datos

Al finalizar las entrevistas, fueron transcritas palabra por palabra y comparadas con la información registrada durante las conversaciones telefónicas. Se revisaron las transcripciones, cotejando el registro en el formulario *on line* (notas de campo) con el audio grabado. Los datos sociodemográficos de los participantes fueron registrados en el programa *Microsoft Excel*.

Para garantizar el anonimato y respetar los principios éticos, las entrevistas fueron codificadas por las siglas: P1, P2, P3…, el número creciente correspondía al orden cronológico de las entrevistas. Además, la información obtenida se utilizó únicamente para los fines de este estudio.

A partir de las transcripciones completas de las entrevistas y para mantener el rigor metodológico, la información fue sometida al análisis de contenido temático de Bardin, realizado en tres etapas, que consisten en: preanálisis (transcripción, constitución del *corpus*, lectura flotante de entrevistas y formulación de hipótesis y objetivos), exploración del material (exportación del material al *software NVivo* ^®^ versión 15, clasificación de datos e incorporación de información, codificación y categorización temática) y tratamiento de los resultados obtenidos e interpretación (articulaciones de inferencias e interpretación de datos, y retomar el marco teórico)^(18)^. También fue valioso aplicar criterios de calidad que involucran exhaustividad, representatividad, relevancia y objetividad^(18)^ para reflejar con precisión las experiencias y realidades de los participantes. Asimismo, como parte del proceso de análisis, se interpretó la percepción de los participantes según la forma en que los entrevistados expresaban sus vivencias y experiencias, además de cómo esas expresiones son interpretadas y categorizadas durante el análisis^(18)^.

También se realizó la interpretación de los datos a la luz de fundamentos teóricos, que se basó en el efecto de la navegación de pacientes en el autocuidado, el conocimiento sobre la enfermedad y el seguimiento con el equipo de salud^(7,10)^. El proceso de análisis de datos consistió en organizar los datos mediante identificación, codificación y creación de categorías. La codificación se realizó después de leer y marcar en la transcripción de las entrevistas, las palabras, frases o temas relevantes para el tema estudiado. Las categorías se formaron a partir de la agrupación de códigos o expresiones que presentaban características similares, por lo que se estableció una relación de complementariedad entre los datos.

A continuación, se clasificaron los datos obtenidos con el fin de lograr la comprensión del texto, dividiéndolo en categorías temáticas emergentes, mediante la organización de expresiones o palabras significativas. Posteriormente se realizó la clasificación e incorporación de información que generaron los principales temas presentados en los resultados. Se llevó a cabo una relectura cuidadosa de las categorías que resumen el contenido de las declaraciones de los entrevistados, que requirió un trabajo intelectual profundo.

### Aspectos éticos

El estudio fue aprobado por el comité de ética de la institución, a través de la Plataforma Brasil, con el número CAAE 20380919800005327, respetando lo establecido en la Resolución 466/2012 del Consejo Nacional de Salud. El investigador siguió la lista de llamadas telefónicas para invitaciones de investigación de la institución que contiene tres opciones para que el participante envíe el Formulario de Consentimiento Libre e Informado (correo electrónico, *WhatsApp* o mensaje), y el documento fue enviado según la preferencia.

## Resultados

La Tabla 1 muestra las características sociodemográficas y clínicas de los 35 (100%) participantes. Llama la atención que 18 (51,4%) fueron hombres; la edad promedio fue de 45 (±13) años y 4 (11,4%) eran fumadores activos.

**Tabla 1 -:** Distribución de los participantes del estudio (n = 35) según variables sociodemográficas y clínicas. Porto Alegre, RS, Brasil, 2022

Característica*	**n = 35**
Edad (años)	45 ± 13
Sexo	18 (51,4)
Masculino	17 (48,6)
Femenino	
Estado civil	23 (65,7)
Casado(a) o en pareja	9 (25,7)
Soltero(a)	3 (8,6)
Viudo(a)	
Fumador activo	4 (11,4)
Ocupación profesional	19 (54,2)
Actividad remunerada	3 (8,6)
Desempleado	13 (37,2)
Jubilado	
Tiempo de diagnóstico	26,9 ±11,4
Hemoglobina glicada (%)	8,0 ±1,2
Comorbilidades ^†^	9 (25,7)
Hipertensión arterial sistémica	6 (17,1)
Enfermedades psiquiátricas	6 (17,1)
Dislipidemia	19 (54,2)
Retinopatía	3 (8,6)
Enfermedad renal por la diabetes	6 (17,1)
Neuropatía sensitiva ^‡^	2 (5,7)
Lesiones en los pies	
Educación	3 (8,6)
Enseñanza primaria	18 (51,4)
Enseñanza secundaria	14 (40)
Educación superior	
Insulina preprandial	6 (17,1)
Regular	29 (82,9)
Análogos	
Insulina basal	15 (42,9)
Protamina Neutra de Hagedorn (NPH)	20 (57,1)
Análogos	

*Variables continuas descritas por media y desviación estándar y variables categóricas por número absoluto y percentil; ^†^Se calculó más de una respuesta para esta variable; ^‡^Se consideró que presentaba neuropatía sensorial cuando el médico la registró en la historia clínica

La Figura 1 presenta las categorías temáticas y los principales temas derivados del proceso de preanálisis y análisis de datos que surgieron de las declaraciones de los participantes.

**Figura 1 -:** Categorías temáticas y temas principales derivados del análisis de las declaraciones de los participantes (n = 35). Porto Alegre, RS, Brasil, 2022

### Manejo de la diabetes

Esta categoría agrupó las percepciones de los participantes sobre la calidad de la atención brindada, el conocimiento adquirido durante las consultas y la mejora en las prácticas de autocuidado como resultado de la atención en salud recibida.

En cuanto a la calidad de la atención brindada, los participantes informaron que estaban satisfechos con el servicio. Las entrevistas que destacaron este tema fueron: P1, P2, P3, P4, P7, P11, P15, P16, P17, P20, P29, P30, P34 y P35. A la mayoría de los participantes les gustó la atención que recibieron y querían que continuara el seguimiento con la navegación de pacientes en la institución. La calidad percibida puede estar fuertemente asociada con las interacciones con profesionales específicos, como las enfermeras que desempeñan un papel central en la atención. Como se destaca en las siguientes declaraciones:

*Felicitaciones por la investigación, ¡me gustó mucho! Me gustó mucho la atención de la enfermera.* (P2)

¡Muy buen*a atención! Me gustaría seguir teniendo algún contacto al que recurrir si tengo alguna duda.* (P7)

¡*Me gustó mucho participar en la investigación! [...], excelente atención [...].* (P11)

Las entrevistas que expresaron opiniones sobre el conocimiento adquirido durante las consultas fueron: P1, P5, P6, P10, P14, P16, P18, P20, P22, P25, P26, P28 y P32. La educación para la salud desempeña un papel importante a la hora de capacitar a los pacientes para el autocuidado, la prevención de complicaciones y adhesión al tratamiento. Algunos entrevistados mencionaron que aprendieron nuevas rutinas de cuidado de los pies durante las consultas, mientras que otros mencionaron la orientación que recibieron sobre las vacunas y el uso de insulina. Se destacan los siguientes extractos:

[...] *realmente me gustó recibir orientación, especialmente sobre las vacunas.* (P1)

[...] *ayudó con cuestiones que nunca habían mencionado los médicos sobre la aplicación de la insulina.* (P14)

*Me gustó aprender a hidratarme los pies y codos.* (P18)

Las entrevistas que mencionaron mejoras en las prácticas de autocuidado como resultado de la atención en salud recibida fueron: P9, P11, P12, P13, P18, P24, P25, P26, P27, P28 y P31. La navegación de pacientes se puede estructurar no solo para proporcionar información sino también para preparar a los pacientes para superar obstáculos e incorporar cambios de manera sostenible. Todas estas entrevistas hicieron referencia a cómo la navegación de pacientes ayudó a los entrevistados a aprender más sobre la diabetes y su manejo. Esas conclusiones surgieron en las siguientes declaraciones:

*¡Me pareció muy bueno! La investigación fue muy esclarecedora, comencé a realizar cuidados que no había hecho antes, creo que quizás por desconocimiento* [...]. (P9)

[...] *es muy válido recordar las cosas que caen en el olvido* [...]. (P11)

*Me gustó aprender nuevas rutinas, mejoré mucho mi cuidado.* (P18)

[...] *He tenido diabetes durante 31 años y siempre estamos aprendiendo algo.* (P31)

Dado que las entrevistas destacaron que la navegación de pacientes contribuyó significativamente a la mejora de las prácticas de autocuidado, especialmente en el manejo de la diabetes, es fundamental analizar cómo la disponibilidad y el formato de los servicios de salud, ya sean remotos o presenciales, afectan esa interacción y continuidad de la atención.

### Servicios de salud remotos y presenciales

Esta categoría agrupó las percepciones de los participantes sobre la disponibilidad de los servicios de salud durante la pandemia, así como el deseo de seguir accediendo a los servicios que brindan las enfermeras y la importancia de la atención recibida.

Las entrevistas que presentaron información sobre la disponibilidad de los servicios de salud para ayudar a autocontrolar la diabetes fueron: P5, P8, P11, P15, P16, P19, P20, P24 y P35. Esos pacientes expresaron su agradecimiento por la viabilidad de la atención que recibieron durante la pandemia de COVID-19 y las medidas de aislamiento social que la acompañaron. La pandemia les impuso desafíos sin precedentes a los sistemas de salud y afectó tanto la oferta como la demanda de servicios, particularmente para las personas con DM, que requieren un seguimiento continuo. Se destacan las siguientes declaraciones:

[...] *quiero agradecerles, me gustó la atención durante la pandemia.* (P19)

*Me pareció muy buena la investigación, me hizo sentir más seguro, me dio seguridad. Agradezco que hayan estado dispuestos a llamarme por la noche porque trabajo durante el día.* (P24)

*Quiero agradecer que me hayan contactado y decirles que su atención fue muy importante, especialmente durante la pandemia, ya que fue una forma de seguimiento durante el período de aislamiento.* (P35)

Los participantes también informaron el deseo de seguir teniendo acceso a los servicios que brindan las enfermeras una vez que finalice la investigación y de forma remota. Las entrevistas en las que se resaltó información sobre al tema fueron: P2, P3, P6, P8, P11, P20, P24, P29 y P33, y las declaraciones de las que surgió el interés de continuar con la navegación de pacientes fueron: P3, P4, P7, P15, P20, P23, P29, P32 y P34. La demanda de los participantes por la continuidad de los servicios remotos indica que es posible que haya un vacío en los sistemas de salud tradicionales. Esa información está presente en las siguientes declaraciones:

*El soporte telefónico y esa forma de atención diferenciada que ustedes brindan debería continuar, si pudiera continuar por* WhatsApp *sería mejor.* (P3)

*Me gustaría que continuara, ya que me ayudó mucho con mi autocuidado, fue muy útil, es genial tener con quién hablar.* (P4)

*Me gustaría que lo mantuvieran. Las consultas presenciales están muy espaciadas y ese servicio remoto supone una forma más de compromiso, que uno va a ser atendido y que tiene que hacer lo correcto. ¡Me gustó mucho que fuera* on line*! ¡Me gustó evaluar los pies!* (P20)

[...] *creo que es imprescindible tener un número de contacto* [...]. (P23)

Las entrevistas que brindaron información sobre la importancia de la atención recibida fueron: P1, P2, P5, P7, P9, P12, P13, P15, P16, P17, P22, P23, P24, P25 y P34. Las mismas abordaron la percepción sobre tener una persona a quien contactar en momentos de duda y cuánto aprendieron con la educación para la salud que les brindaron las enfermeras durante la navegación de pacientes. En contextos de atención a la salud, especialmente para la DM, la relación continua y el apoyo que brindan los profesionales de la salud pueden ser factores determinantes para el éxito del tratamiento y el bienestar del paciente. Como se destaca en las siguientes declaraciones:

*Las consultas me parecieron excelentes, aprendí mucho* [...]. (P5)

[...] *me encantó y la experiencia fue muy buena; me sentí más acogida* [...]*.* (P15)

[...] *aprendí mucho en la investigación, aprendí cosas de las que los médicos no hablan* [...]. (P25)

[...] *muy agradecido por el servicio, la atención y la educación con la que siempre manejó las conversaciones y los exámenes.* (P34)

Sin embargo, un entrevistado manifestó insatisfacción con la atención remota y otro con el intervalo de las consultas. Si bien la atención remota surgió como una solución viable para continuar con el seguimiento de los pacientes en tiempos de distanciamiento social, es posible que la atención no haya sido la misma para todos. Además, el intervalo entre consultas puede afectar no sólo la satisfacción con el servicio, sino también la eficacia del manejo del estado de salud del paciente.

[...] *podría tener un intervalo más corto.* (P11)

*Siempre prefiero la atención presencial.* (P21)

## Discusión

Este estudio buscó comprender las percepciones de las personas con DM1 sobre la calidad de la atención y a través del programa de navegación de pacientes, con el fin de mejorar la atención a las personas con esta condición. Las dos categorías relacionadas con la calidad de la atención que surgieron del análisis de los datos fueron: manejo de la DM y servicios de salud remotos y presenciales.

En el manejo de la DM, los participantes mencionaron que estaban satisfechos con la atención que les brindaron las enfermeras, mediante expresiones como: “Me gustó mucho la atención” y “excelente atención”. Esta percepción positiva coincide con los hallazgos de otro estudio que analizó el aprendizaje de los pacientes sobre la enfermedad y la adherencia al tratamiento^(21)^, que destaca la importancia del vínculo que se establece entre el profesional de la salud y el paciente para el éxito de la atención. Además, el deseo de los participantes de mantener un contacto continuo con las enfermeras sugiere que el apoyo profesional extendido puede ser crucial para mantener las prácticas de autocuidado. La valoración del cuidado y el compromiso de los participantes en la investigación indican no sólo la efectividad de las estrategias utilizadas, sino también la relevancia de brindar un seguimiento continuo y accesible, en el contexto de la satisfacción y continuidad de la atención en salud^(21)^.

En cuanto al conocimiento adquirido durante las consultas, las declaraciones de los participantes destacan lo importante que fue la orientación recibida, especialmente sobre temas que no se abordan ampliamente durante las consultas médicas convencionales. Este tipo de educación para la salud, centrada en aspectos prácticos del autocuidado, es crucial para mejorar este aspecto en personas con DM^(8,10)^. La satisfacción que manifestaron los participantes, al recibir información detallada sobre las vacunas y otras prácticas de autocuidado, sugiere que la orientación que les brindaron las enfermeras llenó vacíos importantes en el conocimiento de los pacientes, lo que coincide con estudios que destacan la importancia de las intervenciones educativas para mejorar el manejo de condiciones crónicas y la promoción de la salud^(8-9)^.

Por lo tanto, la mejora en las prácticas de autocuidado informadas por los participantes del estudio, como la hidratación de los pies, son puntos importantes en el autocuidado de la DM1. Lamentablemente, en Brasil, un estudio realizado en la región sudeste demostró que los entrevistados con DM1 y Diabetes M*ellitus* Tipo 2 (DM2) no llevaban a cabo todas las medidas consideradas de autocuidado de los pies, como inspección diaria, hidratación, corte de uñas, secado de los espacios interdigitales y uso de calzado adecuado. Asimismo, se observó que había una asociación entre menor nivel educativo y menor capacidad para llevar a cabo las medidas de autocuidado de los pies^(22)^. Según un estudio realizado en la India con la misma población, la mayoría de las complicaciones del pie diabético se pueden evitar con educación sobre el cuidado de los pies, evaluaciones periódicas de los pies como estándar de atención y autocuidado como estrategia preventiva^(23)^.

Es importante destacar que la comprensión que tiene el paciente sobre autocuidado es fundamental para controlar exitosamente la enfermedad y, sin duda, prevenir las complicaciones crónicas de la diabetes. Se supone que el autocuidado, cuando se basa en la educación en diabetes y se controla periódicamente, con intervalos más cortos, puede favorecer el desarrollo de un vínculo de confianza entre el paciente y el equipo de salud, y puede aumentar la disposición de la persona con diabetes para que realice el autocuidado. Un estudio transversal con pacientes con DM1, que evaluó la asociación entre conocimiento sobre diabetes y autocuidado mediante cuestionarios, demostró que los participantes con mayor conocimiento sobre la enfermedad mostraron mayor adherencia al autocuidado^(24)^.

En los servicios de salud remotos y presenciales, pero especialmente en los remotos, los entrevistados destacaron la disponibilidad de los servicios de salud para ayudarlos con el automanejo de la DM durante la pandemia de COVID-19. Las personas con DM1, en China, que recibieron seguimiento remoto en el mismo período, presentaron una reducción de los niveles de glucemia en ayunas y de hemoglobina glucosilada (HbA1c), en comparación con los que recibieron seguimiento mediante consulta ambulatoria convencional^(25)^. Asimismo, el automanejo de la diabetes fue evaluado en un estudio realizado en Pakistán durante la pandemia de COVID-19, con 203 pacientes, que utilizó el método de apoyo, valoración, aprendizaje y transferencia de competencias, y presentó las mejores prácticas conductuales y de autocuidado en los pacientes del grupo intervención^(26)^. A pesar de las dificultades que provocó la pandemia de COVID-19 para el tratamiento de la diabetes, la expansión de la atención remota durante este período pasó del 1% al 70%, en algunos centros de salud, lo que posibilitó continuar con la atención y mejorar el control glucémico, el autocuidado y el conocimiento sobre diabetes^(10,27-28)^.

Además, en los servicios de salud remotos y presenciales, ante el deseo que manifestaron los pacientes de seguir accediendo a los servicios que estuvieron disponibles durante el estudio, creemos que es viable implementar la navegación de pacientes para los que tienen DM1 en la institución, dado que es posible reorganizar los recursos humanos existentes en la institución. Por otro lado, este modelo de atención ya existe en otros programas de atención al paciente y se puede adaptar para este público. Los pacientes con diabetes que recibieron seguimiento personalizado, que incluye la navegación de pacientes, mejoraron las tasas de seguimiento con su equipo de salud hasta cuatro veces, realizaron exámenes más frecuentes, mejoraron el control glucémico y el conocimiento sobre la enfermedad y el autocuidado^(8,10,29)^.

A pesar de que hubo importantes avances tecnológicos durante la pandemia de COVID-19, las prácticas de atención en salud en el escenario brasileño siguen siendo un gran desafío para los actuales modelos de atención y gestión. El conocimiento sobre las tecnologías de la información y la comunicación y los nuevos modelos de atención, tanto por parte de los pacientes como del equipo de salud, contribuye a planificar estrategias de atención humanizadas. Reconocer y comprender la percepción de las personas con DM1 sobre la navegación de pacientes durante la pandemia de COVID-19 es un primer paso importante para desarrollar la conciencia clínica sobre este tipo de atención.

Aunque este estudio presenta hallazgos valiosos sobre las percepciones de las personas con diabetes tipo 1 con respecto a la navegación de pacientes durante la pandemia de COVID-19, como ocurre con cualquier investigación, existen limitaciones. Las mismas incluyen que la población estudiada pertenece a un solo centro de atención terciaria de salud, referencia en este tipo de atención y con pacientes de todo el estado. Otra limitación de este estudio es no haberles devuelto a los participantes el texto transcrito de las entrevistas, condición que puede haber afectado los resultados dado el rigor metodológico adoptado. Si bien las entrevistas telefónicas le pueden haber impedido al investigador captar ciertos datos, como el lenguaje no verbal de los entrevistados, permitieron llegar a personas durante el período de aislamiento social y profundizar el conocimiento sobre el tema en estudio en un momento en el que la investigación presencial no hubiera sido posible. Además, ningún estudio brasileño previo estudió la percepción de las personas con DM1 sobre la navegación de pacientes durante la pandemia de COVID-19, una situación excepcional que puede repetirse y para la cual debemos estar preparados.

Además, cabe destacar que el carácter holístico del cuidado de enfermería prepara al enfermero para actuar como vínculo entre los pacientes, los familiares y el equipo de salud, lo que cumple con los objetivos de la navegación de pacientes^(6)^. En otras palabras, los enfermeros se encuentran en una posición favorable para trabajar en diversos sectores y brindar una atención adecuada, eficiente y de calidad. Por lo tanto, este estudio tiene implicaciones significativas para el avance del conocimiento científico en el área de la salud y la enfermería, dado que retrata la percepción de quienes reciben la navegación. De esta manera, contribuye a un campo de conocimiento aún poco explorado y brinda apoyo a futuras investigaciones y prácticas en el área. Invertir en investigación en esta área no sólo mejora las prácticas clínicas, sino que también fortalece el papel de la enfermería como pilar esencial para promover la salud y el bienestar de las personas con DM.

## Conclusión

Ante los resultados, después de analizar las percepciones de los participantes de este estudio, se puedo comprender que la calidad de la atención que brinda el programa de navegación de pacientes es, en general, positiva. La navegación de pacientes benefició a las personas con DM1 en lo que respecta a las prácticas de autocuidado, a tal punto que los usuarios solicitaron que continuara dicho modelo de atención, y vieron una oportunidad más allá de las dificultades y limitaciones que impone la terapia. Además, la realización remota permitió brindar atención personalizada y de calidad en un momento en que no se brindaba atención presencial.

Este estudio también identificó percepciones importantes de las personas con diabetes tipo 1 sobre la navegación de pacientes durante la pandemia de COVID-19. La experiencia y el conocimiento que se genera en tiempos de crisis son fundamentales para desarrollar estrategias de atención más efectivas y humanizadas, que tengan en cuenta tanto las necesidades de los pacientes como las nuevas realidades tecnológicas y organizativas.

Por último, este estudio aporta nueva información para el campo de la investigación sobre la navegación de pacientes con enfermedades crónicas no transmisibles, dado que se centra en las percepciones de los pacientes con DM1, que recibieron ese tipo de atención y expresaron sus percepciones, y que considera la reciprocidad que hay entre las condiciones de salud y los factores contextuales que interfieren en la asistencia que se les brinda a esos individuos en Brasil.
